# Applying a ToF/IMU-Based Multi-Sensor Fusion Architecture in Pedestrian Indoor Navigation Methods

**DOI:** 10.3390/s21113615

**Published:** 2021-05-22

**Authors:** Farzan Farhangian, Mohammad Sefidgar, Rene Jr. Landry

**Affiliations:** Laboratory of Space Technologies, Embedded Systems, Navigation and Avionic (LASSENA), Department of Electrical Engineering, École de Technologie Supérieure, Montreal, QC H3C 1K3, Canada; mohammad.sefidgar@lassena.etsmtl.ca (M.S.); renejr.landry@etsmtl.ca (R.J.L.)

**Keywords:** indoor navigation, time of flight sensor, foot-mounted INS, online calibration, pedestrian navigation, IMU inertial navigation

## Abstract

The advancement of indoor Inertial Navigation Systems (INS) based on the low-cost Inertial Measurement Units (IMU) has been long reviewed in the field of pedestrian localization. There are various sources of error in these systems which lead to unstable and unreliable positioning results, especially in long term performances. These inaccuracies are usually caused by imperfect system modeling, inappropriate sensor fusion models, heading drift, biases of IMUs, and calibration methods. This article addresses the issues surrounding unreliability of the low-cost Micro-Electro-Mechanical System (MEMS)-based pedestrian INS. We designed a novel multi-sensor fusion method based on a Time of Flight (ToF) distance sensor and dual chest- and foot-mounted IMUs, aided by an online calibration technique. An Extended Kalman Filter (EKF) is accounted for estimating the attitude, position, and velocity errors, as well as estimation of IMU biases. A fusion architecture is derived to provide a consistent velocity measurement by operative contribution of ToF distance sensor and foot mounted IMU. In this method, the measurements of the ToF distance sensor are used for the time-steps in which the Zero Velocity Update (ZUPT) measurements are not active. In parallel, the chest mounted IMU is accounted for attitude estimation of the pedestrian’s chest. As well, by designing a novel corridor detection filter, the heading drift is restricted in each straightway. Compared to the common INS method, developed system proves promising and resilient results in two-dimensional corridor spaces for durations of up to 11 min. Finally, the results of our experiments showed the position RMS error of less than 3 m and final-point error of less than 5 m.

## 1. Introduction

Nowadays, indoor localization systems are being used in numerous applications, especially in situations in which the Global Positioning System (GPS) or other signals may have less coverage. Although some indoor positioning systems have been usually based on inertial sensors, in recent decades various sensor fusion methods could increase the accuracy of these systems. Moreover, the significant impact of indoor navigation systems in underground locations, inside multistory buildings, and for pedestrian soldiers cannot be underestimated. In order to satisfy the positioning accuracy of a mobile pedestrian, an adaptive and online calibration method is needed to increase the accuracy of traditional foot-mounted INS algorithms. An online calibration is defined as a process of error estimation for pedestrian’s position, velocity, and attitude, followed by updating the system states in each time-step. The online calibration can restrict the final divergence of the INS. A novel sensor fusion architecture can lead the system to have more accurate error estimation in each EKF or other KF-based models. In this paper, a new sensor fusion method is presented. The architecture of the presented model is based on dual IMUs and a time of flight (ToF) distance sensor. In contrast to the other usual foot-mounted INS methodologies, this method uses a chest-mounted IMU to correct the orientation and heading estimation of a mobile pedestrian. In parallel, another IMU mounted on the foot performs the INS kinematic calculation. Also, the ToF distance sensor is installed on the chest to calculate the distance between the pedestrian and a probable in-front object. An innovative hardware, designed for installing the chest mounted sensors, includes an IMU and a ToF distance sensor. Details of the hardware setup are discussed further.

**Figure 1:**
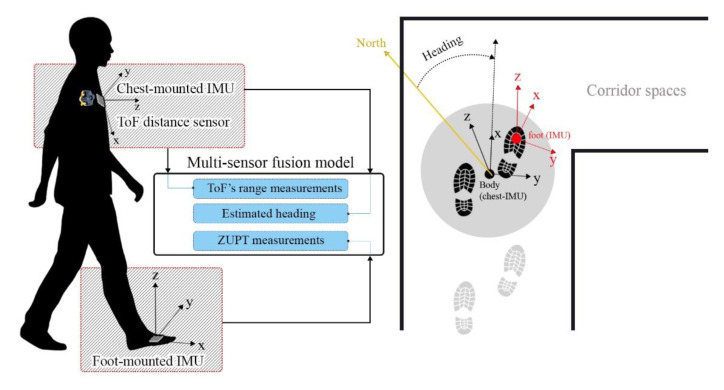
Visual description of installed sensors on the human body, fusion parameters, measurements and orientation of body and sensors.

The proposed system is based on the EKF model, which estimates the errors of position, velocity, orientation, and biases of inertial sensors. The main purpose of the designed sensor fusion architecture is to provide a precise continuous measurement for the EKF estimator in order to calibrate the indoor positioning system. The measurements of orientation are calculated from our last AHRS algorithm, which is used in the EKF system. In this paper, we use the orientation of a pedestrian’s chest instead of a pedestrian’s foot. In other words, due to the large amount of error in attitude and heading estimation while using the foot mounted IMU, an AHRS is accounted for the attitude and heading estimation using inertial and magnetic data of the chest-mounted IMU. As illustrated in Figure 1, the system includes two IMUs. The heading angle of the pedestrian is obtained from a 9-DoF chest-mounted IMU. This IMU utilizes both inertial and magnetic data in order to obtain the chest’s attitude. Moreover, the INS calculates the position, velocity and attitude using a 6-DoF IMU on the foot. In parallel, the IMU on the chest is responsible for calibration of the pedestrian’s orientation and decreasing the heading’s drift. By providing an error between foot and chest orientation, an attitude error measurement could be provided for the calibration system. As well as this, the range measurement of the ToF distance sensor and the zero-velocity measurement of the ZUPT model are considered as main measurements of the system. The designed system extracts the velocity measurements from the range output of the ToF distance sensor.

Since there are various indoor positioning methods using the wireless data transmission systems, these systems can be categorized in the following types: the range measurements using Time of Flight (ToF) distance sensors, Angle of Arrival (AOA) measurements acquired by the antenna, and Received Signal Strength (RSS) data. Numerous previous works have been provided in the next part as a review of literature, however, the main contribution of this paper is fusing the ToF and IMU sensors for online calibration of pedestrian indoor positioning systems.

This article is presented in the seven following sections. In Section 2, we present a complete review of prior works in the literature relevant to our research. Section 3 elaborates an EKF-based AHRS method as well as inertial foot-mounted positioning methodology. In Section 4, we describe the architecture of a proposed multi-sensor fusion method using dual chest-foot mounted IMUs aided by a ToF distance sensor. The experiments and the results are presented in Section 5. Furthermore, the results of all experiments are compared and discussed in Section 6. Finally, the conclusions, as well as some future research potentials, are presented in Section 7.

## 2. Related Works

Until now, there has been a minimal number of attempts to address the problem of pedestrian indoor navigation systems. Many researchers with plentiful algorithms have tried to correct the foot-mounted position estimation in indoor environments. These algorithms utilize the MEMS-based inertial sensors as six or nine degree of freedom (DoF) IMUs consist of three-axis gyroscope, three-axis accelerometer, and three-axis magnetometer mounted on the feet. The theoretical investigation and performance comparisons of various indoor localization systems are included in some surveys. For instance, in [1,2,3], some algorithms such as the Bluetooth-based wireless method, Radar mapping systems, Global Positioning System (GPS)-based methods, and RFID augmentation etc. are comprehensively compared and discussed. The accuracy time, system description, initialization and evaluation methods of each algorithm are well-investigated. Further, the vast majority of technical parameters were included in these articles such as wearability, power consumption, sensor calibration, building model, as well as complexity, cost-effectiveness, and precision.

After presenting the Openshoe method [4] based on the Pedestrian Dead Reckoning (PDR) system with the foot-mounted IMU, and by emerging the Zero-Velocity Potential Update (ZUPT) method in [5], numerous Kalman Filter (KF) and GPS-based indoor localization methods have been presented. For example, online calibration of INS/ZUPT using Extended Kalman Filter (EKF) [6], Constrained Square-Root Unscented Kalman Filter (CSR-UKF) and UKF methods [7,8], magnetic field Gradient-based EKFs [9] are evaluated and discussed. Moreover, various tightly and loosely coupled integrations of indoor PDR systems are designed and evaluated using Bluetooth [10], GPS [11], and Radio Frequency Identification (RFID) [12], which showed a more accurate performance compared to the stand-alone INS and Openshoe methods. In these papers, the localization methods were tested in even Non-Line-Of-Sight (NLOS) cases, in which there are several environmental barriers for the propagated RF signal. The time delay caused by these NLOS emissions are tackled by these works to cope with their estimation and optimization problem. The Bluetooth-based indoor navigation with ToF range sensor fusion could show an efficient performance in real-time embedded implementations. Furthermore, Ultrasonic and Time-of-Flight sensors could present the precision of 1–5 m in harsh environments with more involved geometry [13,14,15]. Although these systems used wireless sensor networks and different accurate measurements, their performances are partially dependent on the building’s complexity, fusion method and, somewhat for Ultrasonic methods, the system cannot locate the pedestrian without the anchors and tags. Further, positioning methods using aforementioned Radio Frequency (RF) signals have the risk of interferences and disturbances, which lead to deterioration of the location error by providing inaccurate measurements.

Accurate attitude and heading estimation using the Attitude and Heading Reference System (AHRS) in indoor positioning algorithms has been one of the main drawbacks which needs to be solved appropriately. Some articles attempted to compensate the heading drift by designing a novel AHRS model. Designing an EKF-based attitude error estimation system [16], attitude accuracy improvement using decision tree [17], and RF signals [18] are discussed and experimented with. In these works, the heading drift compensation is based on the proper antenna and receiver design; however, the methods have been performed in short-term experiments in which their robustness and stability were not proved. Apart from this, map matching methods based on modeling the building architecture using human activity and simultaneous localization and mapping (SLAM) methods can somewhat be effective in 3D positioning in multiple floors and stairways [19,20]. Performance of the PDR systems using the smartphone’s inertial sensors and cameras are also investigated in [21,22]. These methods could not reduce the heading drift in different conditions and trajectories. In these articles, a novel step detection method, stride length estimator, heading inference algorithm, and end-to-end indoor localization system had been designed. These smartphone-based methods were assessed by some in-pocket and in-hand walking scenarios.

Smooth low-speed walking, high-speed walking, multi-floor positioning, and body tracking using the foot-mounted IMUs are well-investigated in the aforementioned articles; however, some systems have not shown fast correspondence to error correction during running experiments. There are various kinds of stance, still, and step detection systems. These detectors are able to compare the statistic metrics obtained from IMU measurements to an especial threshold. Stance Hypothesis Optimal dEtection (SHOE), acceleration-Moving Variance (MV), acceleration-MAGnitude (MAG), and Angular Rate Energy (ARE) are some well-known detectors [23]. Further, the augmentation of the regular foot-mounted INS with a downward-facing range sensor as an Ultrasound-Aided Stance Hypothesis Optimal dEtection (UA-SHOE) is investigated in [23]. These methods showed acceptable position accuracy in walking cases, and more accurate performance during the running experiments. In this paper, we have proposed an adaptive multi-sensor fusion approach for the most prevalent kinds of buildings with straightaways and corridors. By using the distance measurement of ToF range sensors and inertial measurements of two foot- and chest-mounted IMUs, we could achieve highly precise attitude and position estimation. Although some other papers have also pointed out the capability of ToF distance sensors in indoor positioning and mapping methods [24], our design uses the least number of non-inertial sensors and presents a new fusion model integrated with the ZUPT for durable measurement coverage.

## 3. Methodology

### 3.1. AHRS Model for Chest-Mounted IMU

The EKF method with a variable covariance matrix is designed to estimate the quaternion vector of attitude and heading of a chest mounted IMU. The method is based on the magnitude of the angle between the estimated gravitional acceleration, and the measured specific force. This method is completely discussed in our last article metioned in [16]. Also, the error prediction filter regulated by a PI controller is added to diminute the error of attitude and heading estimation in dynamic situations [16]. The EKF model is defined with the state vector $x_{k}={\left[\begin{array}{cc} q_{1} & q_{2} \end{array}\;\begin{array}{cc} q_{3} & q_{4} \end{array}\right]}^{T}$ and the transition matrix $F_{k}$. While, Σ is defined as the rotation angle for each time step, $\omega ={\left[\begin{array}{cc} \omega_{x} & \omega_{y} \end{array}{\;\omega }_{z}\right]}^{T}$ is the angular velocity vector, and dt is the sampling time. The Equations (1) and (2) present the transition matrix in mentioned EKF model.

(1) $$x_{k}{=F}_{k}x_{k-1}{,\;F}_{k}=\cos\left(\frac{\Sigma }{2}\right)I\;+\;\frac{2}{\Sigma }\sin\left(\frac{\Sigma }{2}\right)\left[\begin{array}{c} 0\\ \begin{array}{c} \omega_{x}\\ \begin{array}{c} \omega_{y}\\ \omega_{z} \end{array} \end{array} \end{array}\begin{array}{c} {-\omega }_{x}\\ \begin{array}{c} 0\\ \begin{array}{c} {-\omega }_{z}\\ \omega_{y} \end{array} \end{array} \end{array}\begin{array}{c} {-\omega }_{y}\\ \begin{array}{c} \omega_{z}\\ \begin{array}{c} 0\\ {-\omega }_{x} \end{array} \end{array} \end{array}\begin{array}{c} {-\omega }_{z}\\ \begin{array}{c} {-\omega }_{y}\\ \begin{array}{c} \omega_{x}\\ 0 \end{array} \end{array} \end{array}\right]\mathrm{dt},$$

(2) $$\Sigma =\sqrt{{{(\omega }_{x}\cdot \mathrm{dt})}^{2}{\;+\;(\omega }_{y}{\cdot \mathrm{dt})}^{2}{\;+\;(\omega }_{z}{\cdot \mathrm{dt})}^{2}\;},$$

The normalized specific force, ${\overset{=}{f}}_{b}$, and the normalized horizontal component of the geomagnetic field, ${\overset{=}{m}}_{b}$, are defined as the main measurements of the system, given by Equation (3). The measurement model of the system is highly non-linear, however after obtaining the Jacoban of the measurement matrix, the measurement equation is changed to $z_{k}{=H}_{k}x_{k}$. In which, $H_{k}$ is defined in the Equation (4).

(3) $$z_{k\;}={\left[\begin{array}{cc} {\overset{=}{f}}_{b} & {\overset{=}{m}}_{b} \end{array}\right]}^{T},$$

(4) $$H_{k\;}=\left[\begin{array}{c} q_{3}\\ \begin{array}{c} {-q}_{2}\\ \begin{array}{c} {-q}_{1}\\ \begin{array}{c} q_{1}\\ \begin{array}{c} {-q}_{4}\\ q_{3} \end{array} \end{array} \end{array} \end{array} \end{array}\begin{array}{c} {-q}_{4}\\ \begin{array}{c} {-q}_{1}\\ \begin{array}{c} q_{2}\\ \begin{array}{c} q_{2}\\ \begin{array}{c} q_{3}\\ q_{4} \end{array} \end{array} \end{array} \end{array} \end{array}\begin{array}{c} q_{1}\\ \begin{array}{c} {-q}_{4}\\ \begin{array}{c} q_{3}\\ \begin{array}{c} {-q}_{3}\\ \begin{array}{c} q_{2}\\ q_{1} \end{array} \end{array} \end{array} \end{array} \end{array}\begin{array}{c} {-q}_{2}\\ \begin{array}{c} {-q}_{3}\\ \begin{array}{c} {-q}_{4}\\ \begin{array}{c} {-q}_{4}\\ \begin{array}{c} {-q}_{1}\\ q_{2} \end{array} \end{array} \end{array} \end{array} \end{array}\right],$$

In [16], the initilization process and calculation of the error covariance matrix $P_{0}$ are discussed in detail. The measurement noise covariance matrix, $R_{k}$, which is varied based on the α variable, is calculated in Equations (5) and (6).

(5) $$R_{k}=\left[\begin{array}{cc} \left[\begin{array}{ccc} R_{1} & 0 & 0\\ 0 & R_{1} & 0\\ 0 & 0 & R_{1} \end{array}\right] & 0_{3\;\times \;3}\\ 0_{3\;\times \;3} & \left[\begin{array}{ccc} R_{2} & 0 & 0\\ 0 & R_{2} & 0\\ 0 & 0 & R_{2} \end{array}\right] \end{array}\right],$$

(6) $$R_{1}{=K}_{m}+\alpha {\cdot K}_{n}{,\;\alpha =\cos}^{-1}\left(\frac{\left(C_{n}^{b}g_{n}\right){\cdot \;f}_{b}\;}{\left\|g_{n}\|{\cdot \|f}_{b}\right\|}\right),$$

$R_{1}$ is noise covariance parameter of the specific force designed to vary with time with regard to α, which is the magnitude of the angle between the estimated gravitional acceleration and the measured specific force. $R_{2}$ is the noise covariance parameter of the angular velocity. The $K_{m}$ and $K_{n}$ are regulating constants of the measurement noise covariance matrix. In fact, the constant $K_{m}$ is selected to compensate for the lower magnitude of acceleration, and $K_{n}$ is the weight constant of α. Further, $C_{n}^{b}$ is the transform matrix between the navigation frame to the sensor’s body frame. $f_{b}$ and $g_{n}$ are the estimated gravitional acceleration, and the measured specific force, respectively.

### 3.2. Foot-Mounted Inertial Navigation

**Figure 2:**
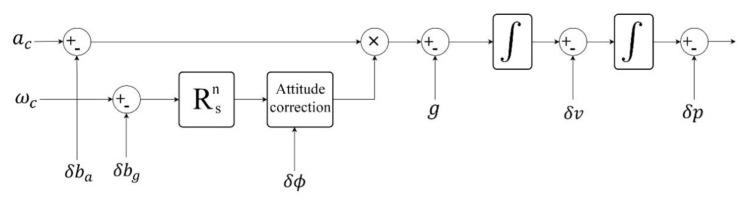
Foot-mounted INS kinematic flowgraph.

The indoor localization system is entirely based on the simple inertial kinematic equations using the outputs of a 3-axis acceleration and 3-axis gyroscope. The overview of foot-mounted pedestrian inertial navigation methods and equations are presented in the following parts. This consists of the bias correction, the rotation matrix integration, the acceleration and gravity removal equation, the position and velocity equations, and calibration of position, velocity, and rotation matrix sections. It should be mentioned that n and s are representing the navigation and body frame indices. The $b_{a}$ is the acceleration bias and $b_{g}$ is the gyroscope drift. The p, v, and a are the position, the velocity, and the acceleration, respectively. Also, $a_{c}$ and $\omega_{c}$ are the calibrated acceleration and angular rate. In addition, $R_{s}^{n}{}_{k}$ is the rotation matrix between body to navigation frame, and g is the earth gravity value. Finally, the vector $\left[\begin{array}{ccc} \omega_{c}{}_{x,k}^{s} & \omega_{c}{}_{y,k}^{s} & \omega_{c}{}_{x,k}^{s} \end{array}\right]$ shows the elements of compensated angular velocity in body frame. Figure 2 represents the foot-mounted INS after performing the online calibration. The EKF-based error states model is introduced in Section 4.3. This model estimates the IMU biases as well as error of position, velocity, and attitude in order to calibrate the INS in each iteration. In the model depicted in Figure 2, the system is calibrated by compensating the position, velocity, and orientation vectors. The following part shows the kinematic equations of foot-mounted INS, see the Equations (7)–(18).

- Bias Correction for acceleration and gyroscope.

(7) $$a_{c}{}_{k+1}^{s}{=a}_{k+1}^{s}{-\;b}_{a}{}_{k}^{s},\;$$

(8) $$\omega_{c}{}_{k+1}^{s}{=\omega }_{k+1}^{s}{-\;b}_{g}{}_{k}^{s},$$

- Rotation matrix integration.

(9) $${\delta \Omega }_{k}=\left[\begin{array}{ccc} 0 & {-\omega }_{c}{}_{z,k}^{s} & \omega_{c}{}_{y,k}^{s}\\ \omega_{c}{}_{z,k}^{s} & 0 & {-\omega }_{c}{}_{x,k}^{s}\\ \omega_{c}{}_{y,k}^{s} & \omega_{c}{}_{x,k}^{s} & 0 \end{array}\right],$$

(10) $$R_{s}^{n}{}_{k+1|k}{=R}_{s}^{n}{}_{k|k}\frac{{2I}_{3\times 3}{+\delta \Omega }_{k+1}\cdot ∆t}{{2I}_{3\times 3}{-\delta \Omega }_{k+1}\cdot ∆t},\;$$

- Acceleration in the navigation frame.

(11) $$a_{c}{}_{k+1}^{s}{=a}_{k+1}^{s}{-\;b}_{a}{}_{k+1}^{s},$$

(12) $$a_{k+1}^{n}{=R}_{s}^{n}{}_{k+1}a_{c}{}_{k+1}^{s}-\;{\left[\begin{array}{ccc} 0 & 0 & g \end{array}\right]}^{-1},$$

- Calculation of position and velocity using Trapezoid integration method.

(13) $$v_{k+1|k}{=v}_{k|k}+\left(a_{k}^{n}{+a}_{k+1}^{n}\right)\frac{∆t}{2},$$

(14) $$p_{k+1|k}{=p}_{k|k}+\left(v_{k}^{n}{+v}_{k+1}^{n}\right)\frac{∆t}{2},$$

- Correction after the online calibration.

(15) $$v_{k+1|k+1}{=v}_{k+1|k}{-\delta v}_{k+1},$$

(16) $$p_{k+1|k+1}{=p}_{k+1|k}{-\delta p}_{k+1},$$

(17) $${\delta \Theta }_{k}=\left[\begin{array}{ccc} 0 & -\phi_{z,k}^{s} & \phi_{y,k}^{s}\\ \phi_{z,k}^{s} & 0 & -\phi_{x,k}^{s}\\ -\phi_{y,k}^{s} & \phi_{x,k}^{s} & 0 \end{array}\right],$$

(18) $$R_{s}^{n}{}_{k+1|k+1}{=R}_{s}^{n}{}_{k+1|k}\frac{{2I}_{3\times 3}{+\delta \Theta }_{k+1}\cdot ∆t}{{2I}_{3\times 3}{-\delta \Theta }_{k+1}\cdot ∆t},$$

The vector $\left[\begin{array}{ccc} \phi_{x,k}^{s} & \phi_{y,k}^{s} & \phi_{z,k}^{s} \end{array}\right]$ is an approximation of the attitude for small angles, and ${\delta \Theta }_{k}$ is its skew symmetric form. In the Equations (7)–(18), k is iteration step and ∆t is the sampling time.

## 4. Multi-Sensor Fusion Architecture

**Figure 3:**
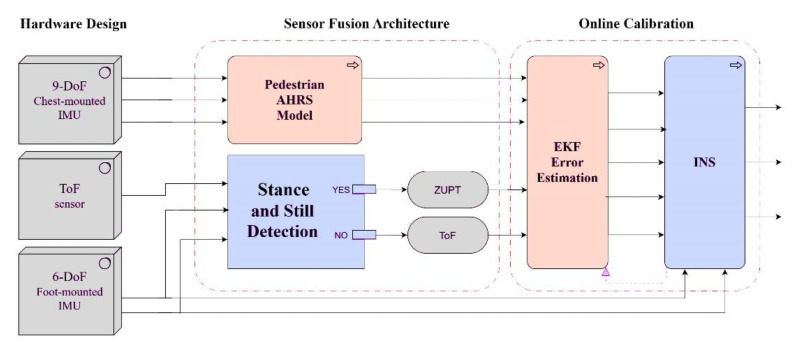
Multi-sensor fusion architecture and online calibration of indoor INS algorithm.

The sensor fusion architecture in this article is based on dual IMU (the chest- and foot-mounted) and a ToF distance sensor. As depicted in Figure 3, there are three types of measurements for the designed EKF error prediction block. The first measurement is obtained from the mentioned AHRS model, which utilizes the orientation of the volunteer’s chest. The second and the third measurements can be used alternatively to compensate the drawback of each other. Because both measurements from ToF and ZUPT are the velocity type, alternative usage of them can lead to the provision of continuous velocity measurements for the EKF estimator. Although the ToF sensor gives only the single direction velocity measurement, it can support the EKF design to estimate more accurate errors. As a result of this, the indoor INS can be calibrated online and persistently. The stance and still detection block is responsible for recognizing the stance steps of the pedestrian. Herein, the ZUPT is performed in stance time and in contrast; when the stance is not detected, the sensor fusion architecture sends the ToF measurement to the EKF system. This part is divided to the four subsections, namely, stance detection phase, corridor detection filter, EKF model and parameters, and measurement model and ToF sensor fusion, respectively.

### 4.1. Stance Detection Phase

**Figure 4:**
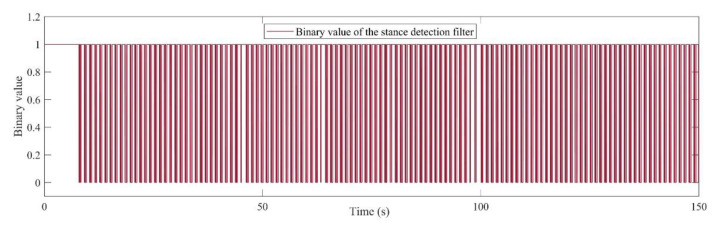
Output of the stance and still detection phase for 150 s walking.

The stance detection phase accounts for distinguishing the times that the pedestrian’s foot is placed on the ground without movement. As depicted in Figure 4, this module can send the binary values for two possible states of the pedestrian, namely, stance (1 value) or movement (0 value). As a result, this phase enables the ZUPT measurements for stance detection, and on the other hand, it can enable the measurement of the ToF range sensor for the EKF estimator. With regard to the stance hypothesis optimal detection (SHOE) algorithm [23], to detect the stance in each time-step, the module should authorize three important conditions. Firstly, the magnitude of acceleration must be between minimum and maximum empirical threshold values. Secondly, the local acceleration variance should satisfy its lower threshold. Finally, the third condition is the magnitude of the gyroscope which should satisfy the special threshold.

In order to detect one stance, all the three of the above conditions, given by Table 1, must be satisfied spontaneously. The recorded data is filtered using the median filter with a window size of at least 11 samples to prevent a faulty stance detection phase. The defined thresholds can be selected in special intervals. The intervals of thresholds for magnitude of acceleration and gyroscope, as well as acceleration variance, are defined in Table 1. The above thresholds can significantly affect the stance detection performance in different pedestrian situations, such as fast walking, slow walking, and running. Further, the amount of the sampling windows can be selected due to the processing speed and desired system accuracy. Figure 4 demonstrates the simple walking experiment at usual speed, which shows the detected stances during 150 s of a pedestrian’s walk. The figure shows the results of steps while all the three discussed conditions are satisfied.

### 4.2. Corridor Detection Filter

Although there are several types of buildings and indoor spaces, most indoor building environments may have passageways connected together with corridors. The main purpose of the corridor detection filter is restricting the heading drift, while the pedestrian keeps walking in the detected corridor. The filter follows the fact that the heading of a pedestrian during a straight walk should not change. The filter is included in the INS block presented in Figure 3. To achieve this, two conditions should be satisfied at the same time, given in Equation (19). The difference of estimated heading between the time steps k and k − w is calculated and compared to an optional threshold $\psi_{\mathrm{th}}$, where w is defined as window size and ψ is the estimated heading.

(19) $$\{\begin{array}{c} \;\left|\psi \left(k\right)\;-\;\psi \left(k\;-\;w\right)\right|\;\leq {\;\psi }_{\mathrm{th}},\;k\;\geq \;w+1\\ \sigma_{k}=\;\sqrt{\frac{\sum_{i=k\;-\;w}^{k}{{(\psi }_{i}\;-\;\mu )}^{2}}{w+1}}\;\leq {\;\sigma }_{\mathrm{th}},\;k\;\geq \;w+1 \end{array},$$

(20) $$\psi \left(k\right)=\psi \left(k\;-\;w\right){\;\mathrm{if}\;\sigma }_{k}\;\leq {\;\sigma }_{\mathrm{th}}\;\mathrm{and}\;\left|\psi \left(k\right)-\psi \left(k-w\right)\right|\;\leq {\;\psi }_{\mathrm{th}},$$

Further, the standard deviation in each window should be less than its threshold defined as $\sigma_{\mathrm{th}}$. μ is the heading’s mean value in one window size. Moreover, Equation (20) shows that the heading in the time step k is equal to the heading in the time step k − w if both conditions are fulfilled. The window size, w, can be determined in a range of 5–20, and $\sigma_{\mathrm{th}}$ is in a range of 70°–90°. As well as this, defining the $\sigma_{\mathrm{th}}$ depends on the window size and can gain a wide range value.

### 4.3. EKF Model, Parameters and Update

The online calibration of indoor inertial positioning systems is based on the extended Kalman filter (EKF) block which estimates the errors of INS. This calibration method utilizes the orientation measurement obtained by an AHRS system connected to the chest mounted IMU. Also, range measurement of the ToF sensor and zero velocity measurement of the ZUPT system are the main measurements of the system, which are switchable by the stance and still detection block. The proposed EKF model has 15 states, which consist of the position error, velocity error, orientation error, and the bias of the gyroscope and the accelerometer. The linearized system model of EKF is determined by the state vector $x_{k}$ as presented in Equation (21).

(21) $$x_{k}{=F}_{k}x_{k-1}{+n}_{k}{,\;x}_{k}={\left[\begin{array}{cc} {\delta p}_{k} & {\delta v}_{k} \end{array}\begin{array}{cc} \delta \phi_{k} & \begin{array}{cc} {\delta b}_{a,k} & {\delta b}_{g,k} \end{array} \end{array}\right]}^{T},$$

where ${\delta p}_{k}$, ${\delta v}_{k}$, and $\delta \phi_{k}$ are the 3-axis error of position, velocity, and attitude. Also, ${\delta b}_{a,k}$ and ${\delta b}_{g,k}$ are defined as the biases of accelerometer and gyroscope, respectively. The transition matrix, $F_{k}$ is presented in Equation (22). The $n_{k}$ is the White Gaussian process noise with covariance matrix $Q_{k}{=E(n}_{k}n_{k}{}^{T})$.

(22) $$F_{k\;}=\left[\begin{array}{c} I_{3\;\times \;3}\\ 0_{3\times 3}\\ \begin{array}{c} 0_{3\times 3}\\ \begin{array}{c} 0_{3\times 3}\\ 0_{3\times 3} \end{array} \end{array} \end{array}\begin{array}{c} \mathrm{dt}{\cdot I}_{3\times 3}\\ I_{3\times 3}\\ \begin{array}{c} 0_{3\times 3}\\ \begin{array}{c} 0_{3\times 3}\\ 0_{3\times 3} \end{array} \end{array} \end{array}\begin{array}{c} 0_{3\times 3}\\ -\mathrm{dt}{\cdot A}_{k+1}\\ \begin{array}{c} I_{3\times 3}\\ \begin{array}{c} 0_{3\times 3}\\ 0_{3\times 3} \end{array} \end{array} \end{array}\begin{array}{c} 0_{3\times 3}\\ \mathrm{dt}{\cdot R}_{s}^{n}{}_{k+1}\\ \begin{array}{c} 0_{3\times 3}\\ \begin{array}{c} I_{3\times 3}\\ 0_{3\times 3} \end{array} \end{array} \end{array}\begin{array}{c} 0_{3\times 3}\\ 0_{3\times 3}\\ \begin{array}{c} \mathrm{dt}{\cdot R}_{s}^{n}{}_{k+1}\\ \begin{array}{c} 0_{3\times 3}\\ I_{3\times 3} \end{array} \end{array} \end{array}\right],$$

where, $A_{k+1}$ is the skew symmetric form of the acceleration vector, and $R_{s}^{n}{}_{k+1}$ is defined as the body-to-navigation coordinate rotation matrix, as formulated in Equations (23) and (24). The orientation is represented in the Euler format as the vector, roll, pitch, and yaw, ${\left[\begin{array}{ccc} \varphi & \Theta & \psi \end{array}\right]}^{-1}$.

(23) $$A_{k}=\left[\begin{array}{ccc} 0 & {-a}_{z,k}^{s} & a_{y,k}^{s}\\ a_{z,k}^{s} & 0 & {-a}_{x,k}^{s}\\ {-a}_{y,k}^{s} & a_{x,k}^{s} & 0 \end{array}\right],$$

(24) $$R_{s}^{n}{}_{k+1}=\left[\begin{array}{ccc} \cos(\Theta )\cos(\psi ) & \sin(\varphi )\sin(\Theta )\cos(\psi )-\cos(\varphi )\sin(\psi ) & \cos(\varphi )\sin(\Theta )\cos(\psi )+\sin(\varphi )\sin(\psi )\\ \cos(\Theta )\sin(\psi ) & \sin(\varphi )\sin(\Theta )\sin(\psi )+\cos(\varphi )\cos(\psi ) & \cos(\varphi )\sin(\Theta )\sin(\psi )-\sin(\varphi )\cos(\psi )\\ -\sin(\Theta ) & \sin(\varphi )\cos(\Theta ) & \cos(\varphi )\cos(\Theta ) \end{array}\right]$$

The estimated states and covariances are updated in each time-step using the Equations (25)–(28). Where, $P_{k}$ is the estimate covariance matrix and $K_{k}$ is defined as Kalman gain. Plus, the $Q_{k}$ and $R_{k}$ are process and measurement covariance matrices, respectively. The $H_{k}$ is the measurement matrix which is completely discussed and formulated in the next subsection.

(25) $$P_{k}^{-}{=F}_{k}P_{k-1}F_{k}^{T}{+Q}_{k},$$

(26) $$K_{k\;}{=P}_{k}^{-}H_{k}^{T}{\;(H}_{k}P_{k}^{-}H_{k}^{T}{+R}_{k}{)\;}^{-1},$$

(27) $$\hat{x}={\;x}_{k}{+K}_{k}\;\left(z_{k}{-\;H}_{k}x_{k}\right),$$

(28) $$P_{k}=\left({I-\;K}_{k}H_{k}\right)P_{k}^{-},$$

### 4.4. Measurement Model and ToF Distance Sensor Fusion

**Figure 5:**
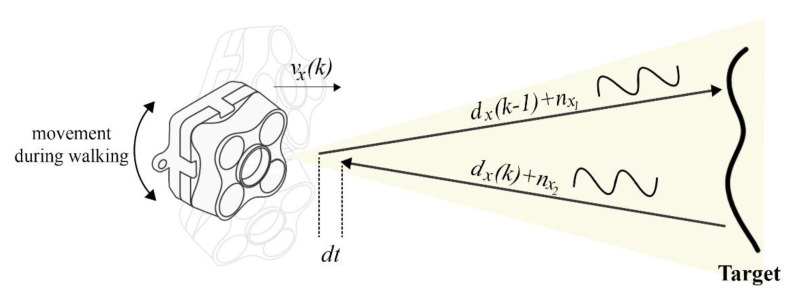
Input and output signals of a ToF sensor in front of a solid target.

The measurement model is defined as $z_{k}{=H}_{k}x_{k}{+v}_{k}$, in which $z_{k}$ is the measurement vector and $v_{k}$ is the measurement noise with covariance matrix $R_{k}{=E(v}_{k}v_{k}{}^{T})$. This model is based on the ZUPT and ToF conditions. Triple conditions are required to use the ZUPT measurements. These conditions are investigated and pointed out in the stance and still phase detection block. Between the detected stance points, there are many times in which the system does not have any measurements. In fact, for a large amount of time, the pedestrian is between two detected steps with no measurement for the EKF system. The main purpose of this fusion architecture is to provide the measurement for these times by using continuous outputs of the ToF distance sensor. Equation (29) shows the velocity model of the ToF output signal, where $F_{s}$ is defined as the sampling rate, $d_{x}\left(k\right)$ is the distance output on the *x*-axis for the time step k, and $v_{x}(k)$ is velocity of the sensor on the *x*-axis for the time step k. Also, $n_{x}$ is the output noise of the ToF sensor considered as the white Gaussian model. Figure 5 illustrates the transmitted and received signals of the ToF range sensor while moving toward a target.

(29) $$v_{x}\left(k\right){=F}_{s}\cdot \left(d_{x}\left(k\right){-\;d}_{x}\left(k-1\right)\right){+n}_{x},$$

To obtain an accurate velocity measurement with the ToF distance sensor, it should be guaranteed that the sensor is installed along the horizontal axis of other IMUs. In fact, this misalignment caused by wrong installation can give the data propagated from other targets which are not along the *x*-axis. Also, the probable noise in output of the ToF sensor has been filtered. Changing the front objects and attitude variation of the ToF sensor can lead to a random noise in output of distance and velocity. The designed filter can restrict the output noise and provide the real measurements of the ToF sensor. Equations (30) and (31) demonstrate the filter’s conditions.

(30) $$\sigma =\sqrt{\frac{1}{N}\;\overset{N}{\underset{j=1}{\sum }}{\left(v_{x}\left(j\right)-\;\mu \right)}^{2}}\;\geq {\;\sigma }_{\mathrm{th}},$$

(31) $$\mu =\frac{1}{N}\overset{N}{\underset{i=1}{\sum }}v_{x}\left(i\right)$$

where N is the size of window and $\sigma_{\mathrm{th}}$ is the standard deviation threshold. Therefore, the measurement model for integration of ZUPT/ToF sensor is formulated in Equations (32) and (33). In the ZUPT mode, the measurements are velocity error ${\delta v}_{k}$, and orientation error $\delta \phi_{k}$ defined as $z_{{\mathrm{ZUPT}}_{k}}=[\begin{array}{cc} {\delta v}_{k} & \;\delta \phi_{k} \end{array}{]}^{-1}$. Further, in the ToF mode the measurement of velocity error is defined for the *x*-axis as $z_{{\mathrm{ToF}}_{k}}=[\begin{array}{cc} {\delta v}_{x_{k}} & \;\delta \phi_{k} \end{array}{]}^{-1}$. The velocity error in the ToF mode can be obtained from subtraction of the ToF measurement and velocity of the INS system. Moreover, $H_{\mathrm{ZUPT}}$ and $H_{\mathrm{ToF}}$ are the measurement matrices for ZUPT and ToF modes, respectively.

(32) $$H_{\mathrm{ZUPT}\;}=\left[\begin{array}{c} 0_{3\times 3}\\ 0_{3\times 3} \end{array}\begin{array}{c} I_{3\times 3}\\ 0_{3\times 3} \end{array}\begin{array}{c} 0_{3\times 3}\\ I_{3\times 3} \end{array}\begin{array}{c} 0_{3\times 3}\\ 0_{3\times 3} \end{array}\begin{array}{c} 0_{3\times 3}\\ 0_{3\times 3} \end{array}\right],$$

(33) $$H_{\mathrm{ToF}\;}=\left[\begin{array}{c} 0_{1\times 3}\\ 0_{3\times 3} \end{array}\begin{array}{c} \left[1\;0\;0\right]\\ 0_{3\times 3} \end{array}\begin{array}{c} 0_{1\times 3}\\ I_{3\times 3} \end{array}\begin{array}{c} 0_{1\times 3}\\ 0_{3\times 3} \end{array}\begin{array}{c} 0_{1\times 3}\\ 0_{3\times 3} \end{array}\right],$$

**Figure 6:**
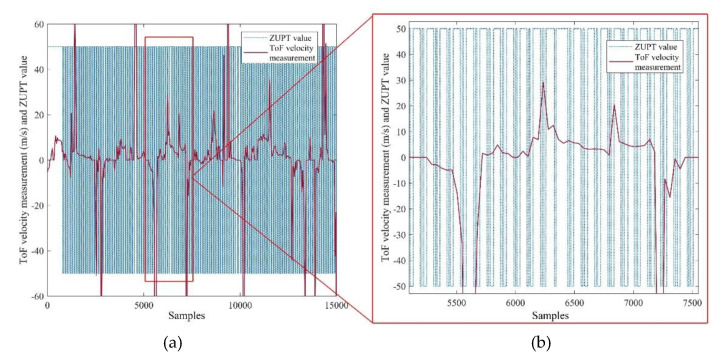
(**a**): Output of the stance and still detection phase and ToF velocity measurement for 150 s normal walking, (**b**): Magnified representation of the figure in 2.5 s.

**Figure 7:**
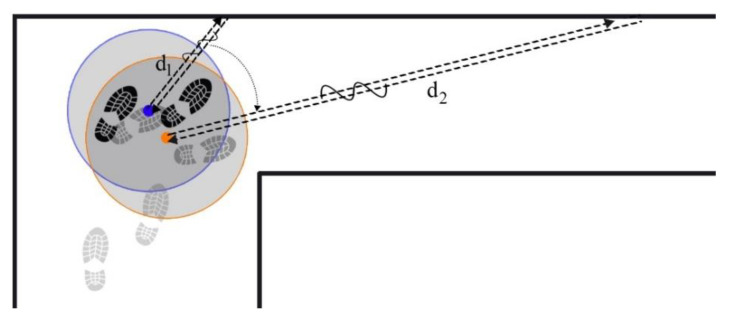
Effect of a significant heading variation in range and, subsequently, velocity measurement of the ToF distance sensor.

Figure 6 illustrates the calculated velocity from the ToF output with a sampling frequency of 100 Hz, and the detected steps which are obtained from the stance and still phase detection block. The velocity is derived from 150 s of walking and 15,000 samples. From the above figure, it can clearly be seen that the ToF sensor in the *x*-axis has provided the velocity measurement. This measurement will be utilized in the times between each detected step, while there is no ZUPT measurement. Contribution of ToF and ZUPT measurements supplies the continuous measurement for the EKF model. In Figure 6b, the positive and negative velocity peaks represent the cases in which the pedestrian turned to another corridor. In these cases, the pedestrian’s heading varied significantly. Further, the measured range measured from the pedestrian to the in-front object increased notably, from $d_{1}$ to $d_{2}$, as depicted in Figure 7.

## 5. Experimental Evaluation

### 5.1. Hardware and Software Setup

This experiment was performed in the corridors of the ETS university by a pedestrian volunteer with a normal walk. The normal walk was considered as the walking of an adult with the average speed of less than 1.4 m/s [25]. The walking path is pre-defined and marked on the building’s map. Further, it had been chosen to have both counter-clockwise (CCW) and clockwise (CW) turns. To perform the highly accurate experiment, an integrated hardware model was designed. The hardware platform consisted of dual nine degree of freedom (DoF) MPU9250 module and a Terarange Evo 60 m ToF sensors. One of the IMUs was mounted on the chest box, and the other one was on the right foot. The chest mounted IMU was located on the designed platform, and to avoid noisy movement of walking, the platform completely tightened to the pedestrian’s body. Table 2 shows the specifications of utilized ToF sensors in the designed platform. The Raspberry Pi 4 was selected to record the IMUs and ToF data at the same time.

**Figure 8:**
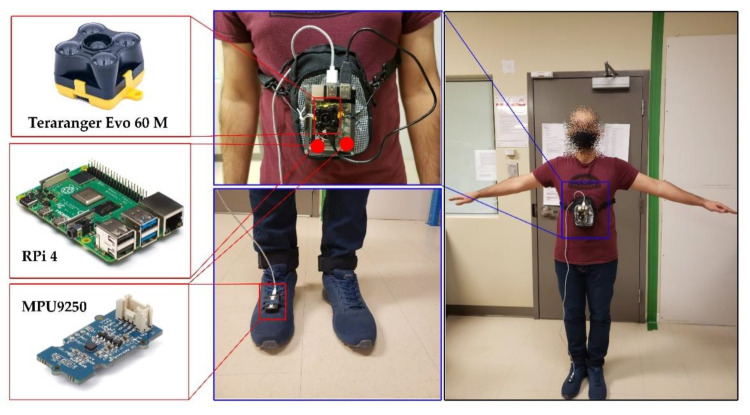
Experimental hardware and software setup.

Both the IMUs and the ToF sensor were used in a data range of 100 Hz. The full-scale range of gyroscopes went up to 2000°/s, and the accelerometer had the scale range of up to ±16 g. The path was approximately 185 m long, and the duration of the experiment was 150 s. The nine DoF data of IMUs was recorded on the Raspberry Pi with a serial transfer, and baudrate of 115,200 bits per second. As the three-axis magnetometer output of MPU9250 is not calibrated, at the first stage the magnetometer data was calibrated. The measured magnetic field data was calibrated using KF-based algorithms, mentioned in [27,28]. Finally, the recorded data of the IMUs and ToF sensor were used for post-processing in the LASSENA laboratory with the MATLAB software. Figure 8 shows the mounted hardware and sensors on the pedestrian’s body.

### 5.2. Experimental Results

Two different scenarios have been designed to evaluate the performance of the proposed indoor navigation system. The goal was to show the accuracy of the architecture in short-term and long-term experiments. Also, the system’s proficiency was analyzed with and without applying the corridor detection filter. In the first scenario, the trajectory was an eight shape. This scenario evaluated the performance of the ToF-aided CDF/INS in a short duration. Moreover, estimated IMU biases, velocity, and pedestrian’s attitude are also discussed in this part. Subsequently, the second scenario focused on the system’s resistance in longer durations. Further, performance of the system was analyzed without the corridor detection filter. Finally, the result of this scenario has been compared with another kind of Kalman-based estimator, namely Unscented Kalman Filter (UKF). Table 3 shows more details about the mentioned scenarios.

#### 5.2.1. First Scenario—Eight Shape

**Figure 9:**
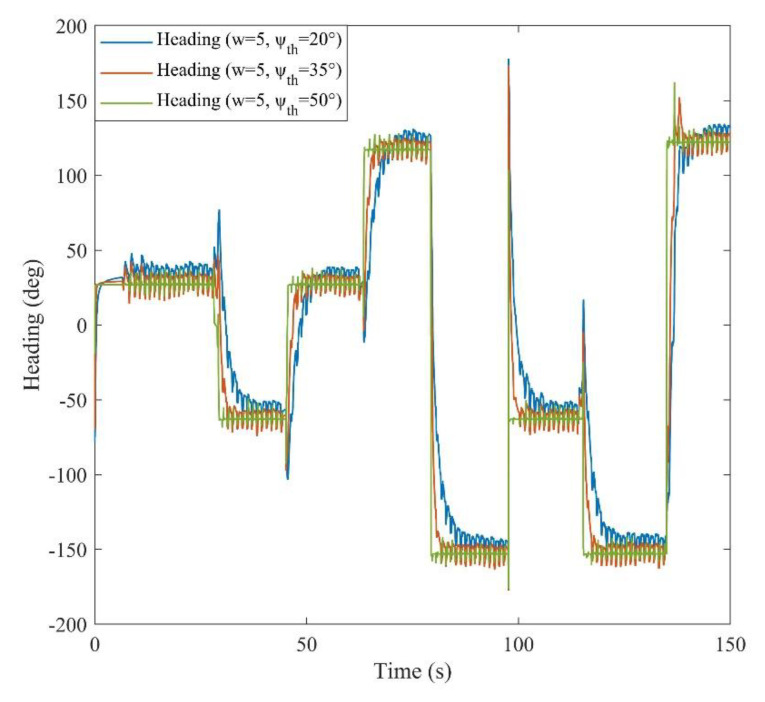
Estimated heading for various window sizes and threshold values.

**Figure 10:**
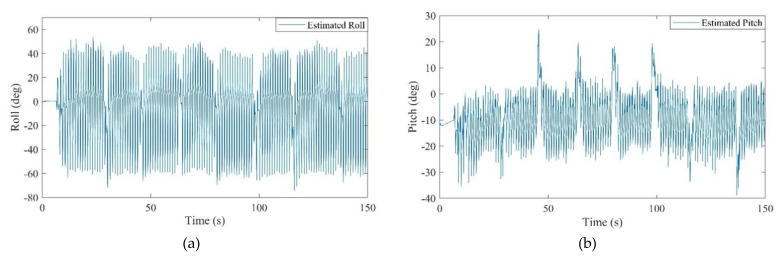
(**a**): Estimated roll angle, (**b**): estimated pitch angle.

After recording the ToF distance measurements and data of dual IMUs installed on the chest and foot, the EKF-based positioning method was implemented. The parameters of corridor detection filter were determined to obtain the most precise heading estimation in each straight walk. The window size and the heading threshold value were adjusted to 5° and 50°, respectively. Furthermore, Figure 9 illustrates the effect of different heading threshold values on the final estimated heading. Further, Figure 10 demonstrates the estimated roll and pitch angles during a 150 s experiment. In the results, it has been shown that by adjusting the proper $\psi_{\mathrm{th}}$, the corridor detection filter was able to keep the heading smoothly constant in each detected straightway.

**Figure 11:**
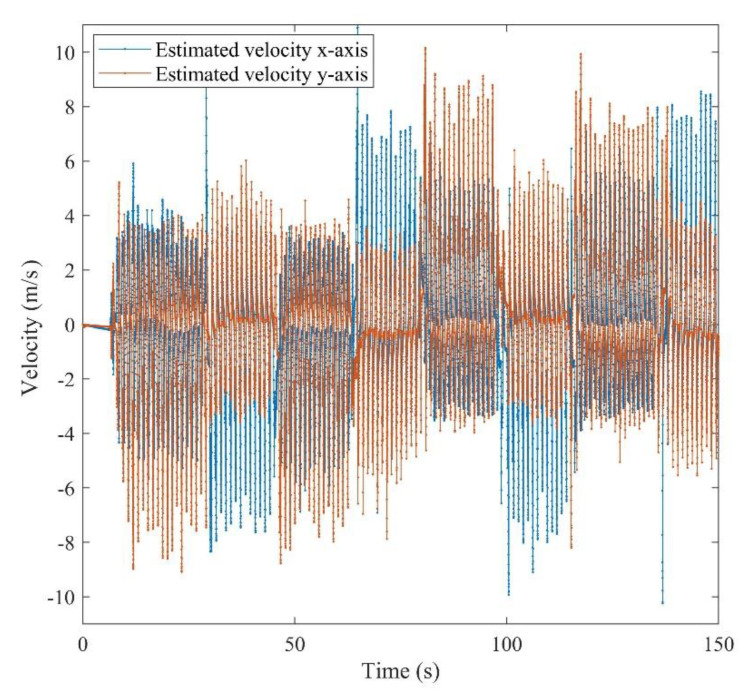
Estimated velocity of the pedestrian for *x*-axis and *y*-axis.

**Figure 12:**
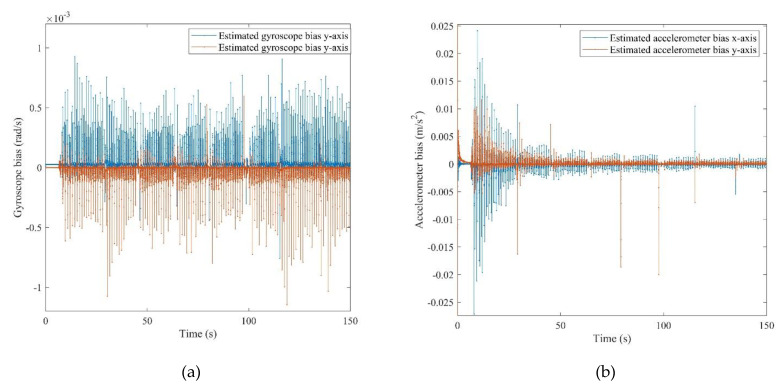
(**a**): Estimated gyroscope bias, (**b**): estimated accelerometer bias for *x*-axis and *y*-axis.

All of the defined states in the positioning system were estimated after the calibration. It should be mentioned that the obtained attitude is a result of the final dead reckoning system using measurements of an IMU which was mounted on the chest. The attitude obtained from the chest measurements outperformed the common foot-mounted INS due to its stability during a normal walk. As can be seen from Figure 10, the peaks and fractures in the roll and pitch angles imply the turn points in the eight-shape trajectory. Figure 11 shows the estimated 2D velocity of the pedestrian during the experiment after the calibration. Moreover, Figure 12 illustrates the estimated bias of the foot-mounted accelerometer and gyroscope.

**Figure 13:**
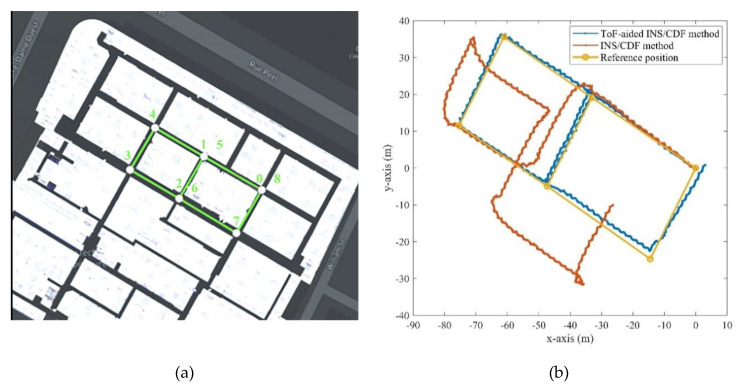
(**a**): The reference trajectory on the map of the building, (**b**): 2D results of the estimated position during the entire trajectory.

As mentioned before, the location of the experiment was corridors of the ETS university. We selected an eight-shape scenario which had seven 90-degree turn points. This scenario can profoundly evaluate the heading estimation and performance of the proposed algorithm in 2D positioning. Since there is only single *y*-axis ToF sensors in this project, and the proposed Corridor Detection Filter (CDF) has not been designed for the multi-floor buildings, a 2D assessment was chosen for the following experiment. Figure 13a shows the selected trajectory with the points marked from 0 to 8. The pedestrian started from the point 0 and finished the route at the point 8, respectively, on a random walk.

Further, Figure 13b illustrates the results of the estimated position on the *x*- and *y*-axis for the dead reckoning INS/CDF method with and without the ToF measurement augmentation. We used a map of the building to obtain the reference key points of the trajectory, which are marked with the yellow points in the Figure 13b. The impact of utilizing the ToF distance measurements is clearly explicit. Although using the CDF system can bound the heading drift after each turn point, measurements of the ToF sensor in its vision area were deemed to be a key measurement for the EKF-based online calibrator.

**Figure 14:**
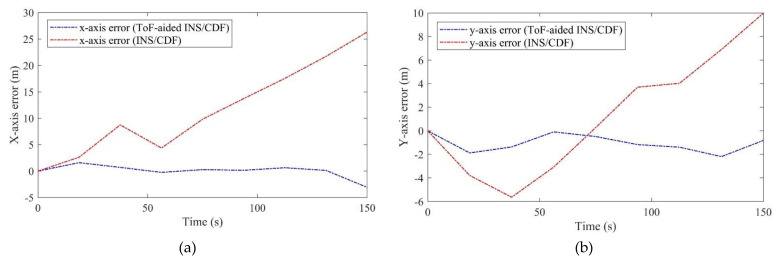
(**a**): The *x*-axis position error before and after ToF augmentation, (**b**): the *y*-axis position error before and after ToF augmentation.

Figure 14 represents the *x*- and *y*-axis errors of the estimated 2D position compared to the true GPS reference. The figure compares the INS/CDF with and without the ToF sensor measurements during the 150 s experiment. We used the GPS true position to initialize the system in both methods. As can be seen, the *x*-axis error started from 0 and culminated to about 25 m; further, the *y*-axis error reached 10 m after 150 s. The position error increased and accelerated more on the *x*-axis. At the end of this part, we will present more details about the error analysis of the proposed method compared to stand-alone INS positioning.

#### 5.2.2. Second Scenario—Rectangular Trajectory

**Figure 15:**
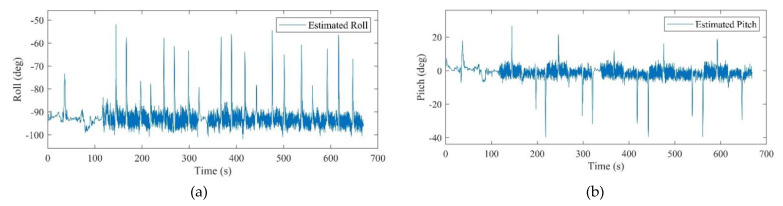
(**a**): The estimated roll for the second scenario, (**b**): the estimated pitch for the second scenario.

**Figure 16:**
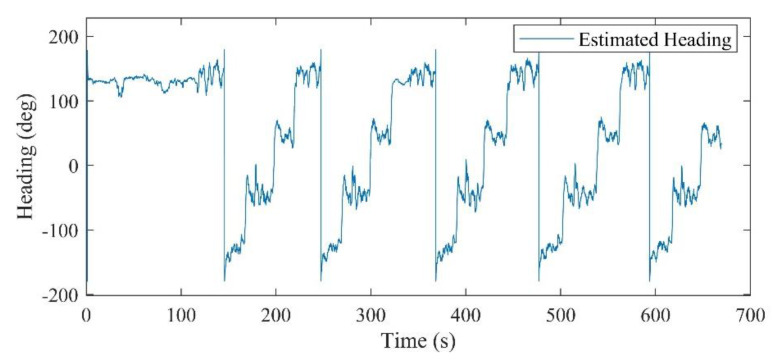
The estimated heading for the second scenario.

**Figure 17:**
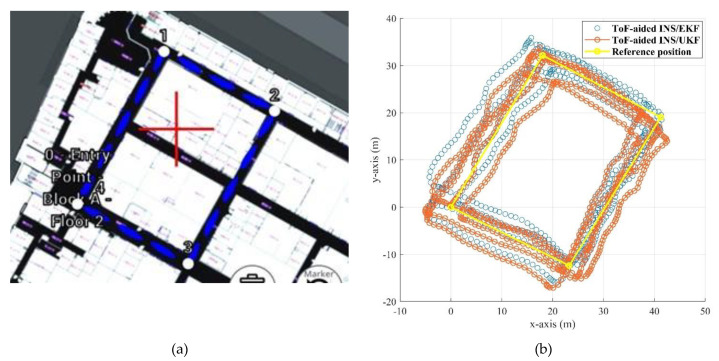
(**a**): The reference map for trajectory of the second scenario, (**b**): the comparison of 2D positioning results for ToF-aided INS/EKF and INS/UKF methods.

All the hardware and software configurations are same as in the first scenario. Inertial and magnetic data of the foot- and chest-mounted IMU were recorded, as well as the measurements of the ToF sensor. The attitude results of the system are demonstrated in Figure 15 and Figure 16. In this experiment, in order to prepare and initialize the system, the walking had been started after 100 s. Figure 17 also shows the positioning result of the system using the proposed ToF-aided EKF system. Performance of the system using the UKF detector has also been shown in this figure. It can be clearly seen that the EKF estimator showed more accurate results during the entire experiment.

**Figure 18:**
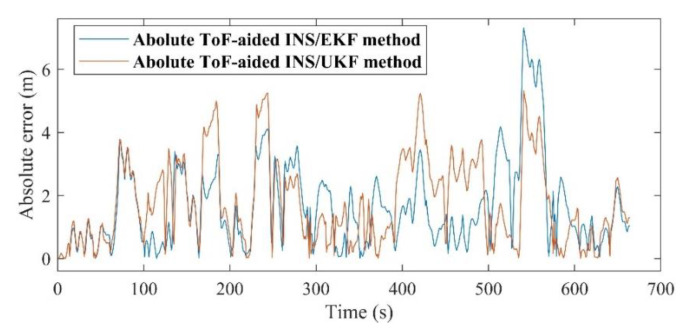
Absolute 2D positioning error.

The absolute maximum error of the EKF is about 7.3 m, while this amount for the UKF is decreased to 5.12 m. Although the RMS errors of both utilized Kalman filters are almost the same, the ToF-aided INS/EKF showed more accurate results during the entire path. Figure 18 shows the comparison of the absolute 2D error of the discussed systems. Finally, the explicit summary of both experiments is listed in Table 4 and Table 5.

## 6. Discussion

There are various points which should be discussed to clarify the proposed method and its practical performance. First, the ToF sensor provides the range measurements in only one axis, which is the direction of walking; however, to have two-dimensional distance and velocity measurements, there is a lack of measurement in the other axis. Nevertheless, due to the presence of the Corridor Detection Filter (CDF), this deficiency was somewhat compensated. Second, to bound the heading drift in the straightaways, the threshold parameters of the CDF should be tuned efficiently. Further, the EKF parameters, such as process noise and measurement noise covariance matrices, should be adjusted effectively. Third, the initialization can also severely affect the final estimated states, although in this research we assumed that the initial position of the volunteer is well-known by the GPS. Fourth, we chose the single-floor eight-shape trajectory, which means that the *z*-axis has not been investigated in our method. Selection of this trajectory is because of the sensor fusion architecture, which is designed to present more errorless results in 2D. In this architecture, the ToF distance measurement contributes only to the *y*-axis. After performing the corridor detection filter, the common dead reckoning INS could estimate the heading more accurately at each turn point; refer to Figure 13. However, as proved in the first scenario, the ToF range measurements have had an important impact on restricting the position error. The end-to-end error in the INS/CDF mode was 28.15 m, which is improved to 3.1867 m after applying the proposed ToF-aided INS/CDF method. Likewise, RMSE of the entire experiment had been decreased from 15.2474 m to a mere 1.7501 m. In addition, the proposed system showed accurate performance for both UKF and EKF nonlinear estimators. Finally, this method could make a significant advancement in the 2D position error. It also showed a considerable effect on decreasing the heading and position divergence. The resistance and stability of the method is tested in the second scenario, while the pedestrian’s position was estimated for about 10 min. The system could show the acceptable performance in both scenarios, and with two different kinds of Kalman filter.

## 7. Conclusions

We have presented an innovative multi-sensor fusion approach for ToF sensor and dual IMU sensors mounted on the chest and the foot. The goal is calibration of foot-mounted indoor positioning systems using range measurements of a ToF distance sensor and MEMS-based IMUs. Various measurements are contributed to provide an accurate INS error estimation. The 9-DoF IMU installed on the chest provides an accurate attitude and heading estimation. Moreover, the INS was implemented using the 6-DoF IMU, fixed on the right foot of the volunteer. The stance and still detection system accounted for step detection using the ZUPT method. Furthermore, an EKF system is responsible for estimating the error states, namely: attitude error, position error, velocity error, as well as gyroscope and accelerometer biases. The main contribution of this paper is providing the consistent velocity measurements using ZUPT/ToF integration. In this case, the ZUPT enables the zero velocity measurements when the steps are detected and its binary value is one. On the opposite side, the ToF sensor provides the velocity measurements for the times that the ZUPT is not enabled. Finally, two experiments were performed to validate the proficiency of the proposed method. In the first experiment, the pedestrian walked in an eight-shape trajectory for 150 s. This experiment evaluated the short-term accuracy of the CDF/INS system. After, for medium-term examination, a rectangular path was selected. In this scenario, the method was tested for 11 min in order to validate its resilience. As a result, the proposed method showed a meaningful positioning accuracy improvement compared to the stand-alone INS and INS/CDF methods. The system was tested with two kinds of Kalman estimators, namely EKF and UKF, to show its compatibility. The end-to-end error reached about 3 m and the RMSE was decreased to less than 2 m for the entire trajectory.

The presented method can be applied to any indoor corridor spaces as an auxiliary augmentation for many kinds of low-cost indoor positioning systems. Although the sensor type and its technical features such as data update frequency, range resolution, detection range etc. can influence the performance, the designed system can provide the velocity measurement from any in-front object. In the future, we will investigate the real-time implementation and on-board calibration of foot-mounted dead reckoning INS, using dual ToF and other vision-aided sensors. Further, the presented fusion architecture can be applied for low-cost indoor localization of ground and unmanned robots in corridor and building environments. Our future purpose is reaching this accuracy for multi-floor trajectories, as well as improving the performance of this system for long-term experiments. Less power consumption, bounding the heading drift, and restricting the position divergence are the main challenges in real-time implementation. Apart from this, the initialization techniques, attitude determination system, and the corridor detection model are our next research topics. Moreover, due to the non-linearity of the state–space model, the high-order EKF models can improve the accuracy of online calibration. Using the Hessian matrix in second order EKF (SOEKF) models can partially increase the positioning accuracy.

## Figures and Tables

**Table 1:** Triple conditions of the stance detection phase.

Number	Condition	Specification	Threshold
1	Magnitude of acceleration	$a_{i}=\sqrt{a_{i,x}{}^{2}{+a}_{i,y}{}^{2}{+a}_{i,z}{}^{2}}$	${\;9\;\mathrm{ms}}^{-2}{\;<\;a}_{i}{\;<\;11\;\mathrm{ms}}^{-2}$
2	Local acceleration variance	$\sigma_{a_{i}}{}^{2}=\frac{1}{2s+1}\overset{i+s}{\underset{k=i-s}{\sum }}{\left(a_{i\;}-\;{â}_{i}\;\right)}^{2}$ ${â}_{i}=\frac{1}{2s+1}\overset{i+s}{\underset{l=i-s}{\sum }}a_{l}$	$\sigma_{a_{i}}{}^{2}{\;>\;9\;(\mathrm{ms}}^{-2}{)}^{2}$ s = 15 (samples)
3	Magnitude of gyroscope	$\omega_{i}=\sqrt{\omega_{i,x}{}^{2}{+\omega }_{i,y}{}^{2}{+\omega }_{i,z}{}^{2}}$	${\;|\omega }_{i}|\;<\;1\cdot 5\;\mathrm{rad}{\cdot s}^{-1}$

**Table 2:** Specification of Evo 60 m Time of Flight (ToF) sensor [26].

Detection Range	Update Range	Weight	Interfaces	Accuracy	Field of View
Up to 60 m	Up to 240 Hz	9 g	USB-2 I2C/UART	±4 cm in first 14 m and 1.5% above 14 m	Approx. 2°

**Table 3:** Details of the experiments.

Experiment	Trajectory	Number of Laps	Total Distance	Duration
Scenario 1	8 shape	1	136 m	150 s
Scenario 2	Rectangular	5	630 m	11 min

**Table 4:** The RMSE and the end-to-end 2D position error for scenario 1.

Method	RMSE	End-to-End Error
Stand-alone INS	221.12 m	375.34 m
INS/CDF	15.2474 m	28.15 m
ToF-aided INS/CDF	1.7501 m	3.1867 m

**Table 5:** The RMSE and the end-to-end 2D position error for scenario 2.

Method	RMSE	End-to-End Error
ToF-aided INS/EKF	1.8369 m	3.3172 m
ToF-aided INS/UKF	2.3456 m	4.1225 m
